# Draft Genome Sequence of Staphylococcus sciuri Strain LCHXa, a Lithium-Tolerant Bacterium Isolated from Laguna Chaxa, Salar de Atacama, Chile

**DOI:** 10.1128/MRA.01549-18

**Published:** 2019-01-24

**Authors:** Claudia Vilo, Camila Salazar-Ardiles, Tamara N. Caimanque, Qunfeng Dong, Nataly Flores, Alexandra Galetović, Jorge E. Araya, Benito Gómez-Silva

**Affiliations:** a Biochemistry Laboratory, Biomedical Department, Faculty of Health Sciences, Universidad de Antofagasta, Antofagasta, Chile; b Center for Biomedical Informatics, Department of Public Health Sciences, Stritch School of Medicine, Loyola University of Chicago, Maywood, Illinois, USA; c Molecular Parasitology Laboratory, Medical Technology Department, Faculty of Health Sciences, Universidad de Antofagasta, Antofagasta, Chile; d Centre for Biotechnology and Bioengineering (CeBiB), Universidad de Antofagasta, Antofagasta, Chile; Broad Institute of MIT and Harvard University

## Abstract

A Gram-positive, coagulase-negative, novobiocin resistant, and lithium-tolerant bacterium was isolated from Salar de Atacama. Strain LCHXa is closely related to Staphylococcus sciuri. Its genome is 3,013,090 bp long and contains 2,551 predicted protein genes. We observed 58 genes associated with stress response and 17 genes linked to osmoregulation, mainly related to glycine betaine metabolism.

## Abstract (precis)

A Gram-positive, coagulase-negative, novobiocin resistant, and lithium-tolerant bacterium was isolated from Salar de Atacama. Strain LCHXa is closely related to Staphylococcus sciuri.

## ANNOUNCEMENT

Reports on the identification and characterization of lithium-tolerant microorganisms are scarce, and most tested microorganisms have a low tolerance to lithium (1–3). This study explored the existence of lithium-tolerant microorganisms at Laguna Chaxa (23°16′53.3ʺS, 68°09′59.64ʺW) in the Salar de Atacama, a basin in northern Chile known as one of the world’s largest lithium reserves (4).

Strain LCHXa was isolated from water samples collected at Laguna Chaxa, Salar de Atacama, and incubated in Luria-Bertani (LB) medium containing 1 M LiCl.

Genomic DNA was isolated from LCHXa cells grown in LB medium using an UltraClean microbial DNA isolation kit (MO BIO) according to the manufacturer’s instructions. The 16S rRNA gene was amplified by PCR using primers 16F27 (5′-CCAGAGTTTGATCMTGGCTCAG-3′) and 16R1525 (5′-AAGGAGGTTGWTCCARCC-3′) and was sequenced in an ABI Prism 377 DNA sequencer. Similarity searches of 16S rRNA genes compared to the NCBI database were done using BLAST, which showed a 96% similarity to Staphylococcus sciuri. Therefore, we assigned strain LCHXa to the genus *Staphylococcus*. Sequencing of the strain LCHXa genome was done with an Illumina MiSeq system. An Illumina Nextera XT DNA sample preparation kit was used for library preparation, and the sequencing was done with a MiSeq V3 600-cycle kit. In total, 3,617,210 reads were obtained, achieving about 350-fold genomic coverage. Evaluation with the FastQC program version 0.10.0 (http://www.bioinformatics.babraham.ac.uk/projects/fastqc/) showed that sequencing produced 300-bp paired-end reads with good quality scores. The A5-MiSeq assembler version 20150522 with default parameters was used for genome assembly that incorporated the steps for data cleaning, error correction, assembly, and quality control (5). The draft genome sequence of LCHXa was 3,013,090 bp long with an average GC content of 32.4% based on 34 scaffolds and with an *N*_50_ scaffold size of 400,359 bp.

**FIG 1:**
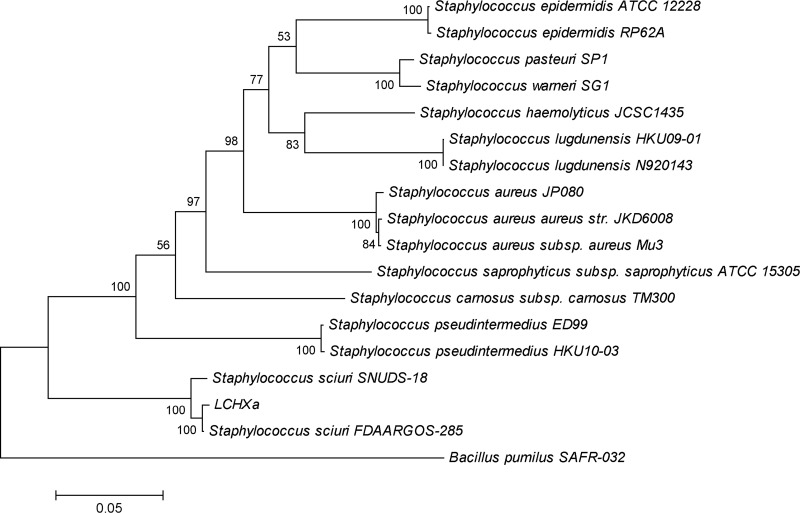
Phylogenetic analysis by maximum likelihood method based on 16S rRNA, *rpoB*, *recA*, *dnaJ*, and *gyrB* concatenated gene sequences. The horizontal bar at the base of the figure represents 0.005 substitutions per nucleotide site. The percentage of trees in which the associated taxa clustered together is shown next to the branches, using a bootstrap of 1,000. Evolutionary analysis was conducted in MEGA7.

The genome was annotated using the Rapid Annotations using Subsystem Technology (RAST) server version 4.0 (6). The isolate of LCHXa had 2,551 predicted protein-coding genes and 65 RNA genes. Among the potential adaptations that help LCHXa to survive in its environmental niches, we observed 58 protein-coding genes associated with stress response and 17 protein-coding genes directly linked to osmoregulation, mainly related to glycine betaine biosynthesis. We used the annotated marker genes, including 16S rRNA, *rpoB*, *recA*, *dnaJ*, and *gyrB*, from strain LCHXa to study the phylogeny. The marker genes were also retrieved from 16 genomes of *Staphylococcus* strains available in the NCBI database. The phylogenetic tree was constructed using a multiple sequence alignment of the concatenated marker genes by ClustalW in MEGA7 (7). The evolutionary history was inferred by using the maximum likelihood method (Fig. 1). The alignment showed an identity of 99% between strain LCHXa and S. sciuri FDAARGOS 258 and an identity of 98% between strain LCHXa and S. sciuri SNUDS 18. Therefore, we assigned strain LCHXa to the species S. sciuri.

LCHXa is the first free-living *Staphylococcus* strain tolerant to molar concentrations of lithium.

### Data availability.

The draft genome sequence assembled from this whole-genome project has been deposited in NCBI under the accession number NADO00000000. The version described in this study is the first version, NADO01000000. The corresponding raw sequencing data sets have been registered in the NCBI Sequence Read Archive under the accession number SRR8238084.
